# Flexible Pressure Sensors Based on Polyvinylidene Fluoride: A Critical Review

**DOI:** 10.3390/ma18030615

**Published:** 2025-01-29

**Authors:** Ming Li, Huaikuan Zang, Jiawei Long, Sijia Sun, Yong Zhang

**Affiliations:** 1 School of Materials Science and Engineering, Hubei Polytechnic University, Huangshi 435003, China; hbpulm@163.com (M.L.); m19007143267@163.com (S.S.); 2 Hubei Engineering Research Center for Collaborative Technology of Advanced Material Manufacturing and Solid Waste Recycling, Huangshi 435003, China; 3 State Key Laboratory of Advanced Technology for Materials Synthesis and Processing, School of Materials Science and Engineering, Wuhan University of Technology, Wuhan 430070, China; zanghuaikuan@whut.ed.cn (H.Z.); ljw168480@whut.edu.cn (J.L.); 4 Center for Smart Materials and Device Integration, Wuhan University of Technology, Wuhan 430070, China

**Keywords:** PVDF, filler, structural design, piezoelectric sensing, sensitivity

## Abstract

With the advent of the intelligent era, flexible piezoelectric tactile sensors, as key components for sensing information and transmitting signals, have received worldwide attention. However, piezoelectric pressure sensors are still currently limited, which severely restricts their practical applications. Furthermore, the demonstrations conducted in labs are not accurate to real-world scenarios. Thus, there is an urgent need to further optimize the intrinsic piezoelectric performance and usage characteristics to meet application requirements. As a representative piezoelectric, polyvinylidene fluoride (PVDF) exhibits significant advantages in terms of excellent flexibility, chemical stability, high electromechanical conversion, low cost, and appropriate acoustic impedance, which allow it to serve as the core matrix in flexible pressure sensors. This paper aims to summarize very recent progress in flexible piezoelectric sensors based on PVDF, including their composition modulation, structure optimization, and applications. Based on a comprehensive summary of recent representative studies, we propose rational perspectives and strategies regarding PVDF-based piezoelectric sensors and provide some new insights for the research and industrial communities.

## 1. Introduction

As one of the most important physiological signals in the human body, touch also serves as the core in intelligent prosthetic hands to perform complex operations and achieve precise external interactions [1,2]. Tactile sensors could capture tactile information and perceive the surroundings, and the high-resolution and precise monitoring of tactile signals (vibration, softness, contact, slippage, texture, etc.) plays an indispensable role in robotics and healthcare [3]. As a typical example, tactile sensors can be used in robotic arms or prosthetics for the disabled, to endow them with some arm ability.

**Figure 1:**
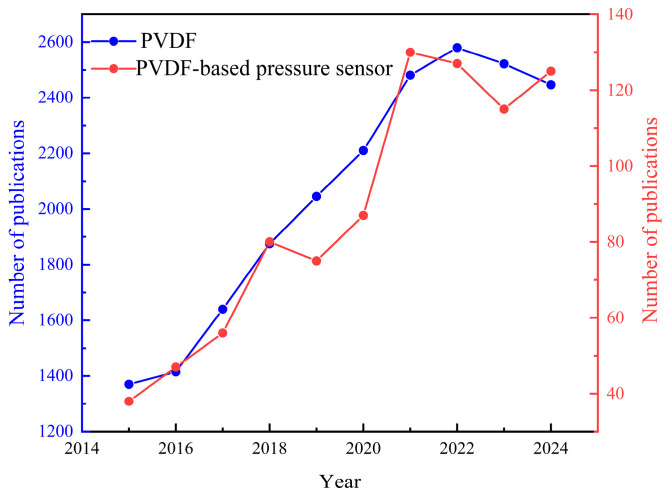
Publications related to PVDF and PVDF-based pressure sensors (data from Web of Science, 27 November 2024).

According to their working principle, pressure sensors can be divided into capacitive, piezoelectric, piezoresistive, and triboelectric types, among others [4,5,6,7,8,9,10]. Among them, piezoelectric sensors present advantages like high sensitivity, wide temperature/pressure measurement ranges, self-powering ability, low energy consumption, etc. As passive sensors, they utilize the piezoelectric effect to convert external mechanical energy into charge, thus realizing the sensing function. As the most common piezoelectric polymer, polyvinylidene fluoride (PVDF) presents excellent piezoelectric and dielectric properties, as well as high flexibility, good processibility, low acoustic impedance, etc. [11,12,13,14,15,16,17]. Typically, their good processibility makes them easy to prepare as flexible piezoelectric sensors of any size and irregular shape, and they are compatible with various processing technologies such as tape-casting, hot-pressing, electrospinning, drawing, etc. [18]. In addition, the crystalline phase of PVDF is significantly affected by the preparation process, and the β-phase exhibits strong piezoelectricity [19,20,21,22,23]. Besides the intrinsic characteristics, various fillers were introduced to further enhance the piezoelectric performance or achieve multifunction. These characteristics make PVDF and its copolymers excellent candidates for flexible pressure sensors. Figure 1 shows the publications related to PVDF and PVDF-based pressure sensors within the last decade. The consistent upward trend from 2015 to 2024 clearly indicates that the research topic is receiving increasing attention due to its great application potential in flexible electronics.

**Figure 2:**
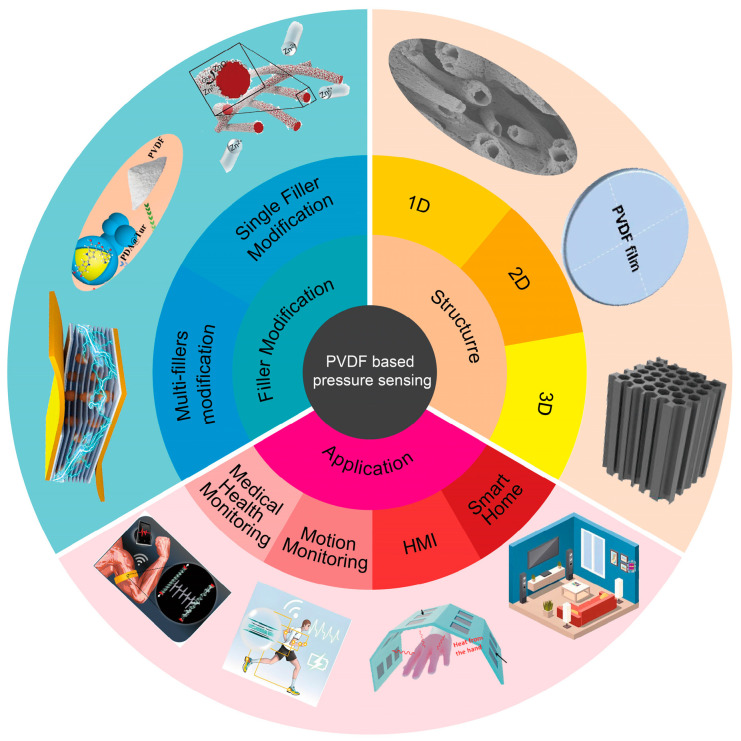
Overview of PVDF-based pressure sensors [23,24,25,26,27,28,29,30,31,32].

This paper focuses on the PVDF and PVDF-based composites used for piezoelectric sensing, comprehensively reviewing the piezoelectric mechanism of pure PVDF and the rational filler doping and structural design for higher sensing capability (Figure 2). Firstly, we discuss the piezoelectricity of PVDF-based composites, as well as the shortcomings of pure PVDF for flexible pressure sensing. Then, we discuss the common and feasible strategies (filler doping and structural design) that are utilized for improving the sensing capability. From the perspective of fillers, single filler and multiple filler modifications were overviewed. The underlying mechanisms of organic polymers, graphite-like fillers, and inorganic nanoparticles as fillers for preparing PVDF-based composites are introduced. In the structural design section, a variety of structures, for example core–shell and hollow structures, etc., utilized for improving the sensing performance of PVDF-based composites are discussed. Furthermore, we summarize the latest applications of PVDF-based pressure sensors, especially in the wearable field. Finally, we discuss the challenges and perspectives for PVDF-based pressure sensing in the near future.

## 2. PVDF and PVDF-Based Pressure Sensors

### 2.1. Piezoelectricity of PVDF

**Figure 3:**
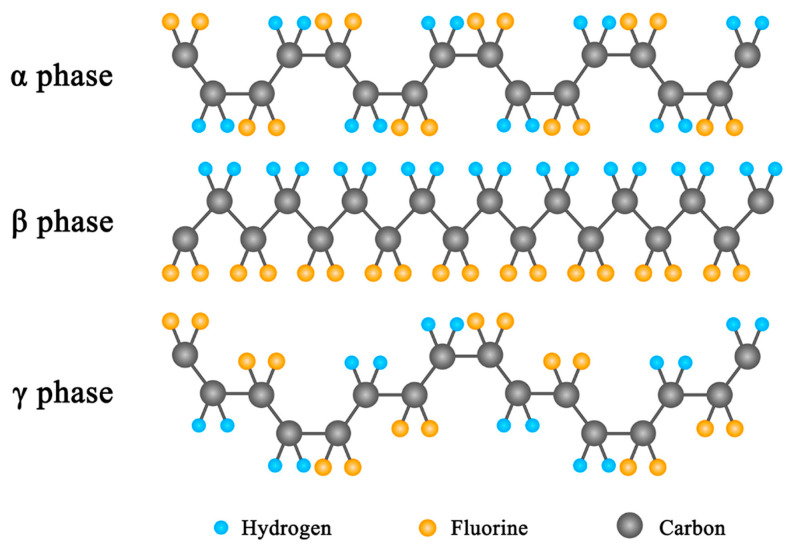
The α, β, and γ crystalline phases of PVDF [33].

Compared to inorganic piezoelectric materials, the piezoelectric coefficient of PVDF and its copolymers is rather low (d_33_ ≈ −20 to −33 pC N^−1^) [33]. However, it can be prepared as fibers, thin films, and porous foams with various morphologies accordingly due to their excellent processability. Moreover, it can easily be composited with various functional fillers to achieve enhanced piezoelectricity [34,35]. There are five crystal phases in PVDF, including α, β, γ, δ, and ε phases [36]. Among them, the α, β, and γ phases are the most common (Figure 3) [33]. The β and γ phases are referred to as the piezoelectric or polar phases, in which two F atoms replace two H atoms that are attached to the same C atom. Due to the repulsion between H atoms and adjacent F atoms, there are no instances where H and F atoms are distributed in the same plane. The -CF_2_ and -CH_2_ groups are arranged alternately, and this tight arrangement provides protection to the carbon chain. As a result, PVDF exhibits excellent chemical and thermal stability [37]. Additionally, the strong polarity of the C-F bond and the alternating arrangement of F and H atoms allow it to withstand ultraviolet radiation from natural sunlight. Due to the strong electronegativity of the F atom, PVDF presents a large dipole moment and thus a high piezoelectric capability. In terms of molecular arrangement, each molecular chain of PVDF contains two possible arrangements: T (trans) and G (gauche, indicating the C-F bond deviates by ±60°). The alternating sequence of the TGTG constitutes the α-phase, which is the most common phase in PVDF. In this conformation, the total dipole moment of the PVDF crystal is zero due to the oppositely directed dipole moments and thus does not show piezoelectricity. Another conformation is the all-trans structure of TTTT (β-phase), in which H and F atoms are on the same side of the molecular chain, causing the dipole moments of the two C-H and C-F bonds to add up and enhancing the polarity [38,39]. Therefore, increasing the β-phase content is key to enhancing the piezoelectricity of PVDF-based composites.

The most common method for nucleating the β-phase is through external field induction to achieve “polarization”, with mechanical stretching or an electric field applied during the nucleation process. Mechanical stretching aids in the transformation from the original spherulitic structure to a crystalline array, wherein molecules are forced to form the polar β-phase, with all dipole moments aligned in the same direction [40]. With an electric field applied on both sides of PVDF, the microcrystalline polar axis would orient along the electric field direction, thus leading to the transformation from α to β-phase [41,42,43,44]. Additionally, the processing strategy serves as an effective way to increase the β-phase content, such as electrospinning for PVDF fibers, phase separation for porous PVDF, and fillers doping for chemically polarized PVDF [45,46,47,48,49,50]. During the electrospinning process, viscous solutions are stretched and then solidified as fibers under a strong electric field, causing the molecular dipoles to orient along the length direction of the fiber, resulting in the α-phase transforming into the β-phase. Chemical polarization refers to adding fillers to the PVDF matrix, inducing the molecular chains to orient around the fillers through the interactions between the filler and matrix molecules, thereby increasing the β-phase content. Furthermore, the mechanical and electrical properties of PVDF-based composites can be improved through structural design [51,52,53,54]. Below, we will discuss the reinforcing effect via filler doping and structural design on the sensing performance of PVDF-based composites.

### 2.2. Modification of PVDF by Adding Filler

#### 2.2.1. Single Filler Modification

As mentioned above, adding fillers into PVDF serves as an effective way to induce chemical polarization and in turn improve piezoelectric performance [55]. The fillers could be small molecules, polymers, and various inorganic nanoparticles. The fillers added to PVDF or its copolymers usually act as nucleating agents, promoting the formation of the β-phase. Furthermore, the size of the fillers also has an impact on the piezoelectricity. To some extent, reducing the size of the fillers helps to increase the interfacial area between the polymer matrix and the fillers, and the interfacial region promotes the coupling effect through the dipole interface layer, thereby generating higher polarization and piezoelectric response.

**Figure 4:**
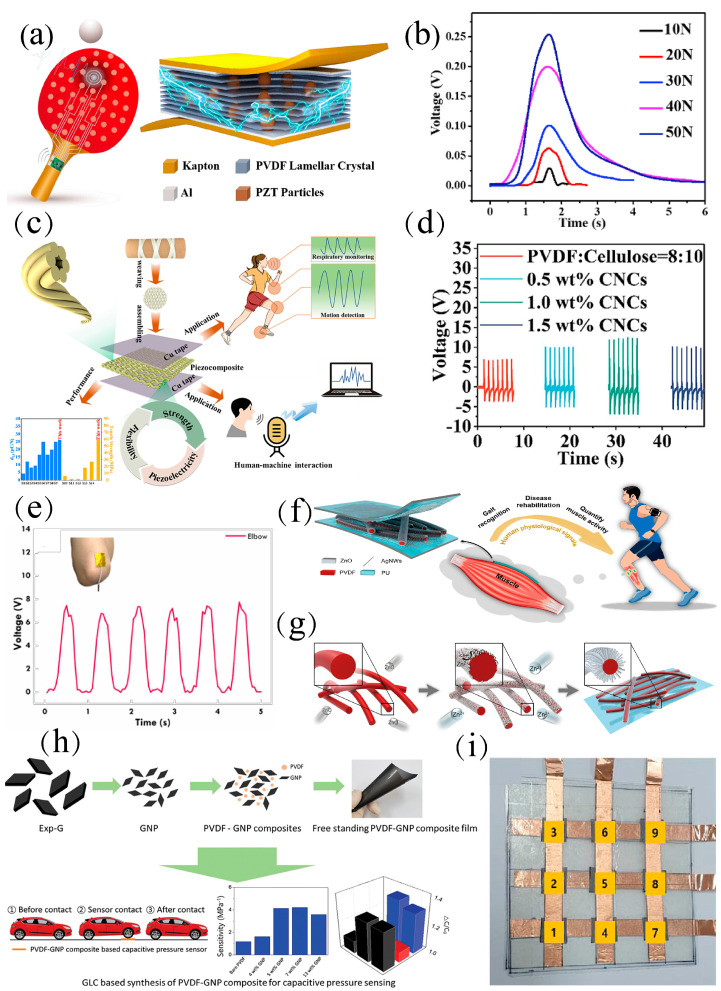
(**a**) Schematic diagram of smart badminton racket for badminton monitoring [24]. (**b**) Output of the sensor [24]. (**c**) Schematic diagram of the piezoelectric fabric [59]. (**d**) Performance of the piezoelectric fabric [59]. (**e**) Limb motion monitoring by PVDF/PCN−based sensor [60]. (**f**) Schematic illustration of the 3D hierarchical interlocked PME based on PVDF/ZnO fibers for muscle behavior monitoring [25]. (**g**) Preparation process of core−shell PVDF/ZnO nanofibers [25]. (**h**) Preparation and application of PVDF−GNP-based composite film [62]. (**i**) Schematic illustration of pressure sensing using a 3×3 sensor array in selected areas [62].

Inorganic piezoelectric fillers serve as a mainstream type to be composited with PVDF for higher piezoelectricity. Lead zirconate titanate (PZT) is the most commonly used piezoelectric ceramic, with a high d_33_ of about 140 pm V^−1^. Guo et al. [24] prepared PZT/PVDF composites with rich flake crystals by the phase separation and hot-pressing process. The piezoelectricity is enhanced due to the potential accumulation effect between each flake crystal and the inorganic particles. Therefore, the prepared piezoelectric composite can show excellent piezoelectricity even at the relatively low doping ratio of 1:10. On this basis, the prepared piezoelectric sensor has a high open-circuit voltage (V_oc_) of 2.51 V, short-circuit current (I_sc_) of 78.43 nA, and ultrafast response time of 21 ms (Figure 4b). The smart ping-pong paddle integrated with 6 × 6 units can map accurate hitting position and impact force in real-time during training (Figure 4a). However, the high lead content (>60 wt%) of PZT is not permitted in the near future [56,57]. Zinc oxide (ZnO) is a typical lead-free piezoelectric semiconductor utilized for mechanical energy harvesting and pressure sensing [58]. Yang et al. [25] proposed an ultrafine, coaxial, aligned, and three-dimensional hierarchically interlocked PVDF/ZnO nanofiber by epitaxially growing ZnO NRs on the surface of electrospinning PVDF nanofibers (Figure 4g). Furthermore, the voltage amplitudes show only a slight fluctuation after 5000 cycles, evidently revealing high durability. The integration of high electrical performance and excellent flexibility was obtained, and the high air permeability of the PVDF/ZnO membrane was verified. In addition, benefiting from the conformal contact between the fibrous membrane and human skin, important physiological signals of the human body including breathing, wrist pulse, and muscle behavior were successfully monitored, thus providing a new strategy for disease rehabilitation, gait recognition, and muscle activity quantification (Figure 4f).

Pan et al. [59] utilized self-polarized PVDF/cellulose composites to prepare flexible fabric piezoelectric sensors (Figure 4c), which exhibited excellent piezoelectric properties (d_33_ = 26.2 pC N^−1^). These sensors could generate an open-circuit voltage of 12 V and a short-circuit current of 100 nA (Figure 4d), which was much higher than previously reported high-voltage poled PVDF piezoelectric sensors. This fabric-based flexible sensor also had the significant features of stable output signal and high sensitivity, thus meeting the requirements for monitoring both tiny and large movements. Chiu et al. [60] introduced phosphorus-doped graphitic carbon nitride (PCN) into PVDF films for self-polarization, thereby improving the sensitivity. The phosphorus atoms were doped into a g-C_3_N_4_ (CN) matrix to enhance the electronegativity of PVDF/PCN composites, thus enabling better attraction of positive charges and exhibiting a pressure sensitivity of 1.48 V kPa^−1^ (Figure 4e).

Adding conductive fillers in the PVDF matrix is well demonstrated as a feasible strategy to improve piezoelectricity and in turn the sensing capability [61]. Kim et al. [62] designed a composite film by compositing PVDF with graphite nanoplatelets (GNPs) (Figure 4h), significantly improving the sensor sensitivity to 4.18 MPa^−1^ in the pressure range of 0.1–1.6 MPa. This enhancement is attributed to the charge generation due to the movement of GNP in the polymer matrix. A 3 × 3 sensor array was designed to realize the accurate detection of specific contact points, thus highlighting their potential for selective pressure sensing (Figure 4i). Studies have shown that graphene oxide (GO) has polar interactions with PVDF, resulting in trans-conformation of PVDF chains [63,64]. Lee et al. [65] prepared PVDF/GO fibers by dry jet wet spinning technology, which presents much higher mechanical strength than that of pristine PVDF. With only 0.05 wt% CNT in aligned PVDF hollow nanofibers, the sensitivity could be increased from 450 mV N^−1^ to 940 mV N^−1^, an increase of about 108% [66].

Organic polymers, i.e., hydrocarbons and their derivatives, have high molecular weights. When they are doped with PVDF, the -CH_2_ and-CF_2_ groups of PVDF form hydrogen bonds and other interactions with C=O and-NH_2_ groups, thereby increasing the β-phase content. Xiong et al. [67] successfully prepared a PVDF/dopamine (DA) membrane using electrospinning technology. The unique structure significantly improves the piezoelectric performance, with high sensitivity (7.29 V N^−1^, 0–4 N) and excellent durability (10,000 cycles). In addition, the potential application of this PVDF/DA film as a flexible wearable sensor for monitoring human motion and subtle physiological signals was also verified. Liu et al. [68] prepared a PVDF/polyacrylonitrile (PVDF/PAN) film by the electrospinning method. The morphology of the PVDF/PAN film is relatively uniform, with most fiber diameters ranging from 100 nm to 300 nm. At the same time, the β-phase dominates in this film (i.e., the β-phase content is up to 83.4%), and its tensile strength and elongation are about 7 MPa and 26%, respectively. The output voltage of 1.3 V was obtained under a tiny impact force of 1 N. Moreover, it shows good application prospects in real-time monitoring of human motion (such as jumping, running, walking, finger bending, etc.).

#### 2.2.2. Multi Fillers Modification

Incorporating multi-fillers into the PVDF matrix to enhance piezoelectricity is a newly emerging strategy in recent years [69]. On the one hand, its purpose is to optimize the mechanical properties of the composites through the synergistic effect of multi-fillers, meeting the processing and usage requirements. Additionally, multiple conductive fillers were involved to improve the electric field distribution or electrical conductivity within the matrix, enabling effective charge immigration [70].

**Figure 5:**
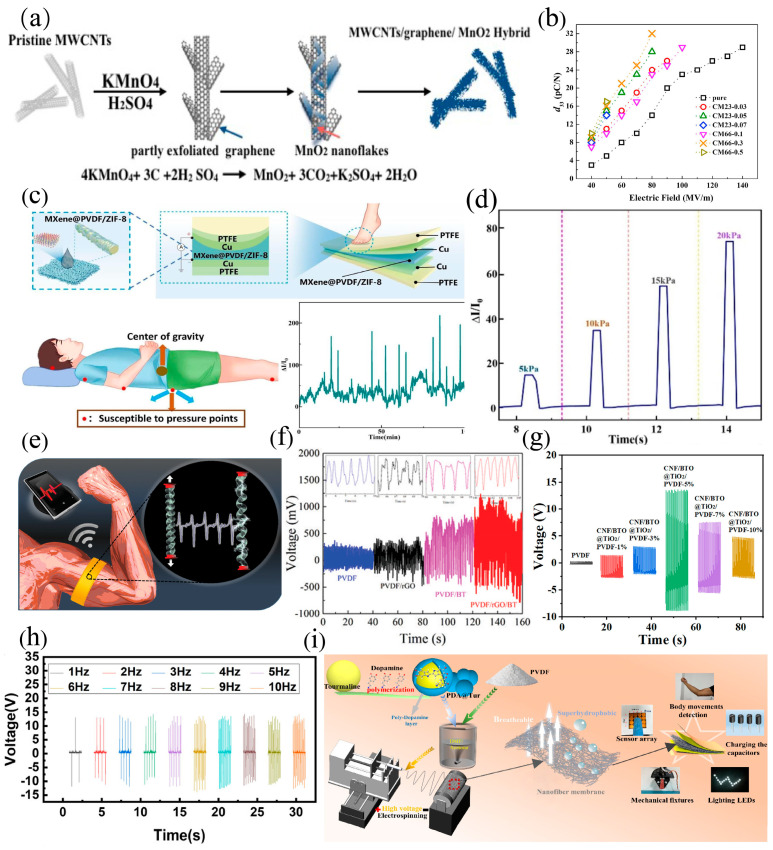
(**a**) Synthesis route of MWCNTs/graphene/manganese dioxide hybrid material [71]. (**b**) Piezoelectric coefficient of the composites under different electric fields [71]. (**c**) Structure of MXene/PVDF@ZIF−8 sensor and its application in human body monitoring [72]. (**d**) Performance of the pressure sensor made of MXene/PVDF@ZIF−8 [72]. (**e**) Schematic diagram of the charge generation during the longitudinal extension process of PVDF/rGO/BT nanocomposite coil [26]. (**f**) Electrical output of PVDF nanocomposite when extending the axial 1 cm coil structure [26]. (**g**) Performance of CNF/BTO@TiO_2_/PVDF−based pressure sensors [73]. (**h**) Performance of PDA@Tur/PVDF PENG [27]. (**i**) Schematic diagram of the sensor preparation [27].

Graphene and carbon materials possess excellent conductivity, high specific surface area, and good dispersibility, making them ideal fillers to be involved in PVDF for higher mechanical and sensing capability. Yang et al. [71] proposed a simple strategy for constructing three-dimensional hybrid nanostructures composed of manganese dioxide/graphene/multiwalled carbon nanotubes (MnO_2_/Graphene/MWCNTs) (Figure 5a) to improve the piezoelectric properties of PVDF. By adjusting the content of MnO_2_, the breakdown strength and piezoelectric properties can be modulated accordingly. Under a relatively low polarization electric field (50–80 MV m^−1^), the nanocomposites can obtain a high piezoelectric coefficient of 17–33 pC N^−1^ (Figure 5b), which is twice that of PVDF. Mokhtari et al. [26] designed a series of wearable sensors using hybrid PVDF/reduced graphene oxide (rGO)/barium titanate (BT) fibers with distinct structures (Figure 5e). The coiled PVDF/rGO/BT fibers were stretched to ≈100% strain, generating a peak power density of 3 W Kg^−1^ (Figure 5f), which was 2.5 times higher than previously reported piezoelectric textiles. Gao et al. [72] prepared MXene/PVDF@ZIF-8 multilayer fiber membranes through in situ electrospinning technology, in which ZIF-8 particles were introduced to construct the rough morphology (Figure 5c). Furthermore, MXene nanosheets were introduced into PVDF@ZIF-8 nanofibers by vacuum filtration technology, to construct a conductive network with a multidimensional structure along the vertical direction of the film. The pressure sensor has a wide working range (0–125 kPa), high sensitivity (25.8–77.9 kPa^−1^), a fast loading/unloading time (20/30 ms), and good mechanical stability. A variety of human movements and lying postures were recorded with precise feedback, proving the broad application prospects of flexible sensors in wearable devices (Figure 5d).

BaTiO_3_, as a typical lead-free piezoelectric, is often utilized as the filler in PVDF-based composites. Zhang et al. [73] synthesized a novel core–shell structured cellulose nanofiber/BaTiO_3_@TiO_2_(CNF/BTO@TiO_2_) with the assistance of bio-macromolecular CNF. The introduction of CNF could optimize the dispersion of BTO in the water phase and enhance the integrity of the core–shell structure. Due to the interfacial space polarization generated between the TiO_2_ shell and BTO core, the core–shell structure could promote the polarization of electric dipoles and the formation of the β-phase in PVDF. When the content of the core–shell structure was 5 wt%, the β-phase content reached 61.89%, and the piezoelectric coefficient was up to 84.29 pm V^−1^. As a result, the maximum open-circuit voltage (V_oc_) and short-circuit current (I_sc_) of the piezoelectric composites were as high as 13.10 V and 464.3 nA (Figure 5g). In addition, its excellent pressure-sensing capability and durability make it a good candidate for various flexible electronic devices. Wang et al. [74] used polydopamine@BaTiO_3_ (pBT) nanoparticles as interlayer bridges to construct an interlocked interlayer interface that could covalently bond each component layer through a simple and scalable method. The obtained film exhibits significantly improved piezoelectricity (a maximum piezoelectric coefficient of 27.2 pC N^−1^), power density of 1.72 μW cm^−2^, and stability over 8000 cycles. This self-powered device could be used to detect a variety of physiological activities and could be scaled up for manufacturing eco-friendly, flexible, and durable self-powered wearable sensors.

Furthermore, environmentally friendly biopolymer polymers are also a common type of filler that is incorporated in PVDF. Yang et al. [27] utilized the unique multifunctionality of tourmaline nanoparticles (Tur) to achieve highly enhanced piezoelectricity by incorporating polydopamine (PDA)-modified Tur in PVDF (Figure 5i). The PDA@Tur nanofillers not only effectively increased the piezoelectricity of PVDF but also played an important role in optimizing the wettability, elasticity, and air permeability of the piezoelectric sensor. The maximum output voltage reached 31.0 V (Figure 5h), which was four times that of the pure PVDF counterpart. At the same time, the sensitivity reached 0.7011 V kPa^−1^ within 1–10 N, which was 3.6 times that of the pure PVDF film (0.196 V kPa^−1^). The piezoelectric coefficient was up to 5.5 pC N^−1^, which was much higher than that of the pure PVDF (3.1 pC N^−1^). The output signal diagrams corresponding to clapping, finger, knee, and elbow movements were detected, with a response/recovery time of 24/19 ms.

### 2.3. Structural Design

In addition to the incorporation of fillers, structural design serves as another mainstream strategy for improving the sensing capability of PVDF-based pressure sensors [75,76,77,78,79,80,81,82,83]. In addition, 1D core–shell and hollow structures, 2D thin film structures, and 3D foam stereoscopic structures, as well as the construction of various surface microstructures, have been proposed and utilized in PVDF-based pressure sensors [84,85,86,87,88].

#### 2.3.1. One-Dimensional Structure

**Figure 6:**
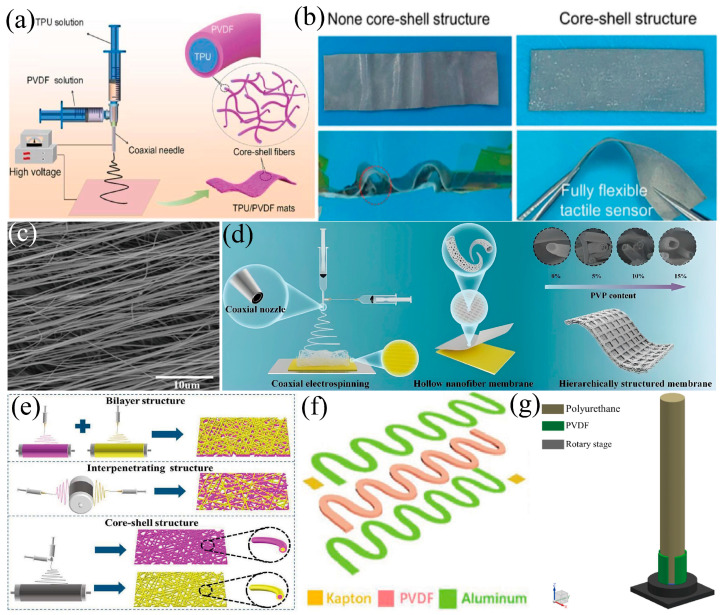
(**a**) Schematic illustration of the fabrication of core–shell structured piezoelectric mat [89]. (**b**) Photos of non-core–shell and core–shell structured sensors after cyclic loading [89]. (**c**) FE−SEM micrograph of highly aligned PVDF/CNT nanofibers [66]. (**d**) Schematic diagram of the fabrication process for micro-structured hollow PVDF nanofibrous membranes [28]. (**e**) Schematic of the fabrication process [90]. (**f**) Schematic of a PVDF device [92]. (**g**) Schematic of a PVDF array [93].

The core–shell structure refers to a composite structure in which the central part (core) and the peripheral part (shell) are composed of different materials. The introduction of a core–shell structure can improve the sensitivity, cyclic stability, and piezoelectric performance through the stress concentration effect. Common preparation processes include electrospinning, spin-coating, etc. [77]. Wei et al. [89] constructed a pressure sensor with a TPU/PVDF piezoelectric pad (core–shell structure) as the sensitive layer and a TPU/AgNWs pad as the flexible electrode (Figure 6a). This design not only enhances the stretchability of the sensor but also maintains excellent piezoelectric capability, endowing the sensor with high sensitivity (20.3 mV N^−1)^ and good durability (Figure 6b). Even under deformation, this tactile sensor can correctly respond to mechanical stimuli, and successfully monitor various pressures generated in daily life. In some studies, doping fillers and structural design are utilized simultaneously to construct the PVDF pressure sensors. Zhang et al. [90] prepared PVDF-based composite membranes doped with different mass fractions of MXene and ZnO by electrospinning and assembled them into flexible self-powered piezoelectric sensors. The addition of MXene and ZnO endows PVDF membranes with better piezoelectric activity. PVDF/MXene-PVDF/ZnO membranes have a double-layer structure (DL), interpenetrating structure (I), and core–shell structure (CS), as shown in Figure 6e. Through the synergistic effect of filler doping and structural design, the piezoelectric properties of PVDF-based membranes can be further improved. Among them, the output voltage has a good linear relationship with the applied pressure and can produce a high piezoelectric response to the bending deformation caused by human motion. In addition, Li et al. [18] used a simple strategy to fabricate core-sheath piezoelectric fibers (C-PEF) by directly electrospinning PVDF onto stainless steel wires. This C-PEF can respond well to different degrees of bending deformation, so it can be assembled as a bending sensor to monitor human sleep behavior.

Hollow structures were proposed to improve the sensitivity and energy-harvesting efficiency of PVDF-based sensors [91]. Zhang et al. [28] propose an innovative method to fabricate hollow PVDF nanofibers with a hierarchical structure by coaxial electrospinning technology, as shown in Figure 6d, for enhancing the sensing capability. The nanofibers present high β-phase content (91.31%), excellent flexibility (maximum strain 56.9%), and high porosity (88.94%), enabling the sensor to exhibit a high sensitivity of 2.7 V N^−1^ (1.08 V kPa^−1^) and a maximum output voltage of 10.1 V, which was approximately three times that of solid PVDF nanofibers without microstructures. In addition, the sensor could maintain a stable output even after 14,400 cycles and was successfully applied for human motion monitoring and gesture recognition, showing a fast response (60.4 ms) and the ability to control mechanical hands to perform different gestures synchronously. Sharafkhani et al. [66] prepared ultrathin-shelled PVDF/CNT nanocomposite with aligned hollow fibers, showing an increase of about 250% in output voltage and about 18% in β-phase content compared with random solid nanofibers (Figure 6c). Besides the above-mentioned hollow and core–shell structures, there are many other 1D structures for special purposes. Guo et al. [92] designed a PVDF-based stretchable sensor with a serpentine layout, as shown in Figure 6f, which improved the sensitivity and realized the dynamic monitoring of speed, angle, joint motion, and environmental vibration. Wei et al. [93] designed a piezoelectric energy harvester with an axially symmetrical distributed PVDF array, for two-dimensional vibration energy harvesting and direction sensing (Figure 6g). Therefore, we can design and prepare PVDF-based pressure sensors with novel structures through various advanced technologies according to the requirements of actual application scenarios.

The electrospinning technology faces practical issues such as the preparation of demanded spinning solutions, adjustment of spinning parameters, and controlled environmental factors when manufacturing core–shell and hollow structures of PVDF piezoelectric materials. Optimizing these conditions serves as the core for obtaining the desired morphology and piezoelectric response.

#### 2.3.2. Two-Dimensional Structure

**Figure 7:**
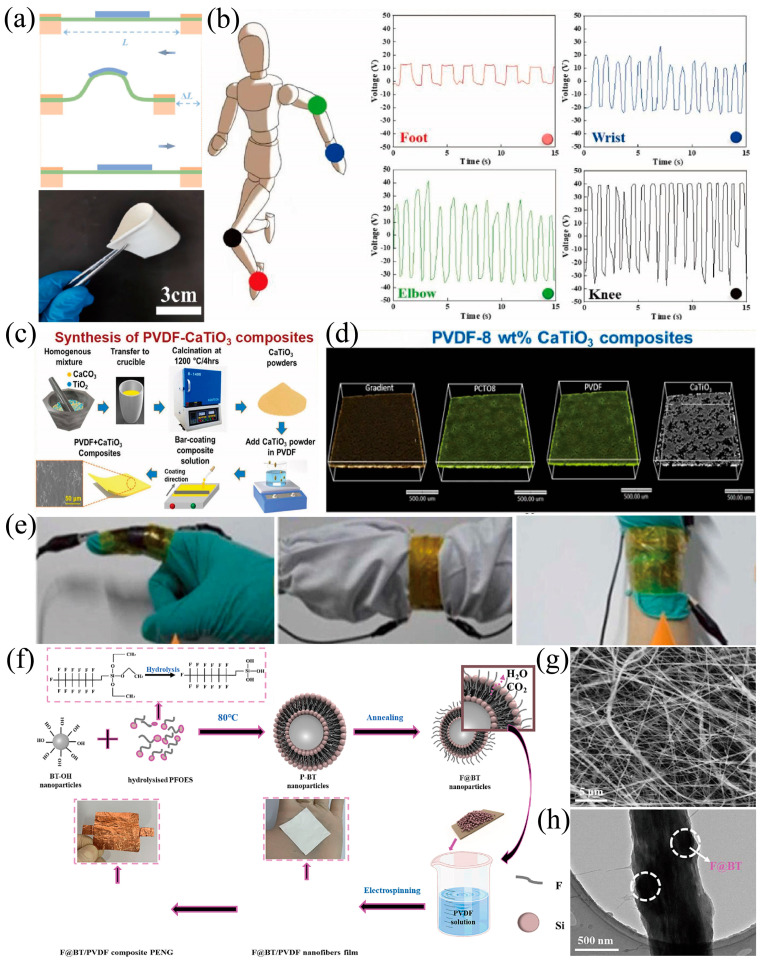
(**a**) Schematic diagram of the film deformation process and its digital photos [94]. (**b**) The voltage output generated by PEH attached to different parts of the human body [94]. (**c**) Synthesis methodology of CTO particles and PVDF−CTO composites [95]. (**d**) X−ray tomographic 3D visualization of PVDF−CTO composite film at 8% [95]. (**e**) Human body signal collection picture [96]. (**f**) Preparation process of F@BTPVDF composite PNG [97]. (**g**) SEM image of F@BTPVDF composite nanofibrous membrane [97]. (**h**) TEM image of F@BTPVDF composite nanofibrous membrane [97].

Thin-film structure is another common type in PVDF-based flexible pressure sensors. Compared to 1D structures, 2D-structured PVDF-based pressure sensors are more suitable for wearable devices and flexible electronics. Zhang et al. [94] achieved a significant improvement in piezoelectric energy harvesting efficiency and sensing capability by constructing a polymer-impregnated nanoparticle network within a cellulose scaffold. Three different methods were proposed to regulate the microstructure of the composite film, which shows good flexibility (Figure 7a). It was found that when the cellulose content is 3 wt%, the electromechanical coupling efficiency is optimal. The maximum power density is up to 42 μW cm^−3^, an increase of nearly 800% compared to their traditional counterpart (Figure 7b).

CaTiO_3_ (CTO) powder was synthesized by solid-state reaction and mixed with PVDF matrix to prepare flexible composites with thin-film structure (Figure 7c) [95]. The uniform dispersion of CTO particles in the PVDF matrix was confirmed by X-ray tomography images (Figure 7d). It was found that the electrical output increased with the addition of CTO, with the optimal performance (power density of 0.19 μW cm^−2^) obtained when the CTO content is 8 wt%. The device was mounted on the heel, to sense the skipping exercises and detect skipping patterns through digital signal processing techniques and artificial neural network (ANN).

Cao et al. [96] grew ZnO on exfoliated MoS_2_ nanosheets by in situ polymerization, and then doped it into PVDF to prepare PVDF/MoS_2_@ZnO piezoelectric films. The effects of different contents of ZnO on its structure, mechanical energy harvesting, and sensing performance were discussed emphatically. ZnO was deposited on the surface of MoS_2_ nanosheets to form heterostructures (MoS_2_@ZnO), which can effectively promote the transformation of α-phase to β-phase in PVDF. Thus, the β-phase content was increased from 6.2 ± 2.5% to 54.3 ± 2.7%. It also exhibited excellent mechanical properties, with a tensile strength of 37.64 ± 5.12 MPa and an elongation at a break of 9.77 ± 1.57%. The flexible piezoelectric sensor with a thin-film structure could precisely collect pressure signals caused by human body movement (bending of fingers, wrists, and elbow joints) (Figure 7e).

Wang et al. [97] prepared a core–shell structured barium titanate (F@BT) nanoparticle by using fluorine-containing silane coupling agents to modify the surface of BT nanoparticles, followed by thermal annealing (Figure 7f). The core–shell structured design improved the dispersion of BT nanoparticles in the PVDF matrix (Figure 7g,h). In turn, the β-phase content and piezoelectricity in the F@BT/PVDF composite film were improved dramatically.

#### 2.3.3. Three-Dimensional Structure

**Figure 8:**
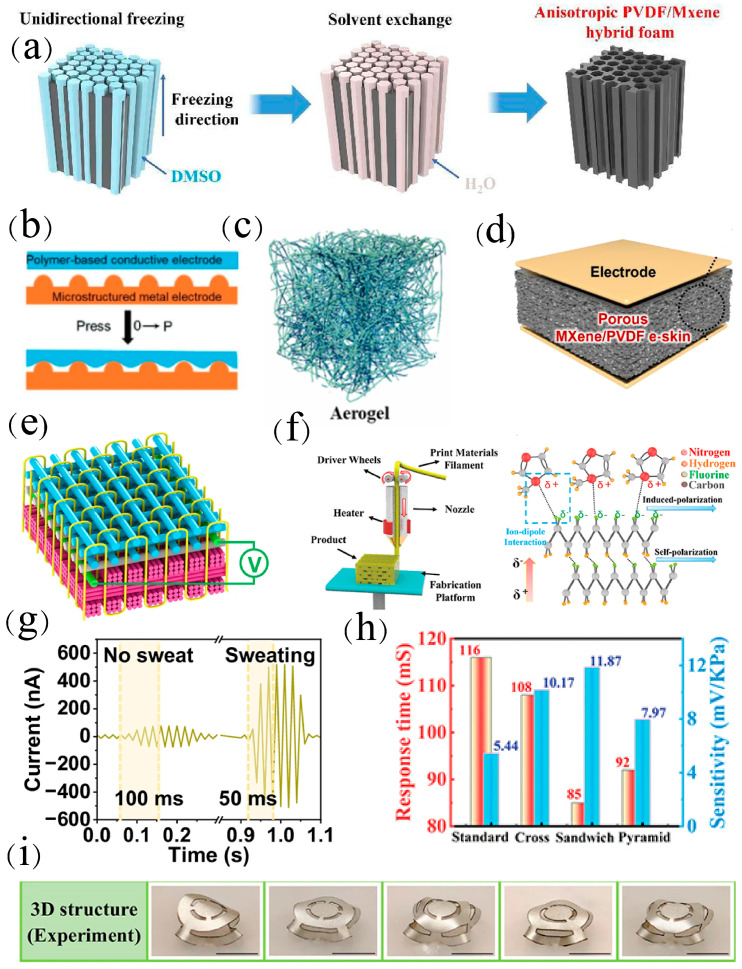
(**a**) Schematic diagram of the manufacturing process of PVDF/MXene composite foam structure [29]. (**b**) Schematic diagram of sensor microstructure [103]. (**c**) Aerogel of three−dimensional structure [100]. (**d**) Mxene/PVDF−based porous foam [98]. (**e**) Schematic diagram of three-dimensional fabric structure [101]. (**f**) Performance comparison of 3D fabric sensors with and without sweat [101]. (**g**) Voltage sensitivity of 3D fabric sensors [101]. (**h**) FDM 3D printing process and structural schematic diagram [102]. (**i**) Response time and sensitivity of four different structures [102].

Three-dimensional structures generally present high specific surface areas, which may endow the materials with unique electrical properties. Han et al. [29] successfully constructed anisotropic PVDF/MXene devices with directional micropores through delicate formulation design for the first time (Figure 8a). The anisotropic foam was used as a directional sensor with the current as the sensing signal, and the sensitivity displayed in the direction perpendicular to the microchannel was 3.93 times that of its orthogonal direction. In addition, the foam exhibited the highest sensitivity of 41.3 nA kPa^−1^ in the pressure range of 2.5–20 kPa, which was superior to most piezoelectric composite sensors. This novel strategy provides a practical way to construct water-insoluble polymer-derived devices with anisotropic structures in a green and energy-saving manner. Kim et al. [98] designed a self-powered piezoelectric sensor with high sensitivity and a wide sensing range based on the 3D porous MXene/PVDF (Figure 8d), which was able to monitor high-frequency dynamic signals such as sound waves and low-frequency radial arterial pulses. In particular, it could detect high-frequency vibrations generated by sliding friction and could sense various surface textures with different roughness and modulus, which well demonstrated its great application potential in wearable devices, prostheses, robots, and medical monitoring equipment.

Zhang et al. [99] initiated a strategy to regulate sensing performance by utilizing different 3D structures and encapsulation materials with varying Young’s moduli (Figure 8j). The relationship between aspect ratio (α), pattern factor (η), the elastic modulus of the encapsulation material, and the equivalent stiffness was obtained through finite element simulation, providing theoretical guidance for the design of 2D precursors and the selection of encapsulation materials. This structural design offers a strategy for performance control of piezoelectric pressure sensors. Sun et al. [100] employed a wet electrospinning process to construct a novel PVDF aerogel with a 3D structure and controllable super-elasticity (Figure 8c). This 3D aerogel features adjustable thickness, a 3D porous structure, and ideal mechanical properties. The 3D hierarchical porous structure endows the aerogel with notable electrical performance and outstanding mechanical strength, with optimal voltage and current output of 156 V and 34.9 μA. Furthermore, this pressure sensor can detect human motion, showing broad application prospects in self-powered pressure sensing.

Commercial wearable piezoelectric sensors are electronically packaged and almost airtight, thus reducing the comfort of the human body. To address this issue, Fan et al. [101] developed a PVDF nanoyarn with an intensity as high as 313.3 MPa and used advanced 3D textile technology to weave it with different yarns into a 3D piezoelectric fabric (3DPF) sensor (Figure 8e). Among the reported flexible piezoelectric sensors, this reported 3DPF has the highest tensile strength (46.0 MPa). The 3DPF has an antigravity unidirectional liquid transport function that allows sweat to move from the inner layer near the skin to the outer layer within 4 s, providing a comfortable and dry environment for users. Sweating does not weaken the piezoelectric performance of 3DPF, but rather enhances it (Figure 8f). In addition, the durability and comfort of 3DPF are similar to those of commercial cotton T-shirts. This work provides a novel strategy for developing comfortable wearable electronic devices. Liu et al. [102] proposed an innovative strategy of combining 3D printing with rational structural design (Figure 8h) to construct customized 3D-printed piezoelectric lattice units. They show fast response time, high sensitivity, and excellent linearity over a wide pressure range, outperforming most of the reported thin-film pressure sensors (Figure 8i). Three-dimensional printing technology provides powerful tools for modeling and operating complex 3D piezoelectric pressure sensors, which can be used for intelligent sensing applications that cannot be achieved by traditional technologies.

The design of surface microstructures is also very helpful for improving sensing capability. Kong et al. [103] proposed a piezoresistive pressure sensor with high sensitivity and ultra-large sensing distance based on inelastic metal microstructures and elastic flat PDMS/PVDF films (Figure 8b). During the pressure loading process, due to the rigidity characteristics, the metal microstructure continuously penetrates into the polymer film, thus resulting in continuous changes in the contact area and resistance. The pressure sensor has an ultra-large pressure sensing range, high sensitivity, excellent sensing distance (300 kPa for PDMS and 1.4 MPa for PVDF), ultrafast response time (≈3.5 ms), and high stability (>10,000 cycles). Acceleration sensors were constructed based on the fabricated pressure sensors, which are widely used in motion recognition, virtual reality, and vehicle safety systems. This work provides an alternative strategy for constructing high-performance pressure sensors.

## 3. Applications

PVDF-based flexible pressure sensors have a wide range of applications, including health monitoring [30,104,105,106,107,108,109], sports monitoring [31,110,111,112,113], human–machine interaction(HMI) [114,115], smart homes, and others. Recently, some special scenarios have been proposed. A comprehensive summary will be given below.

### 3.1. Medical Health Monitoring

**Figure 9:**
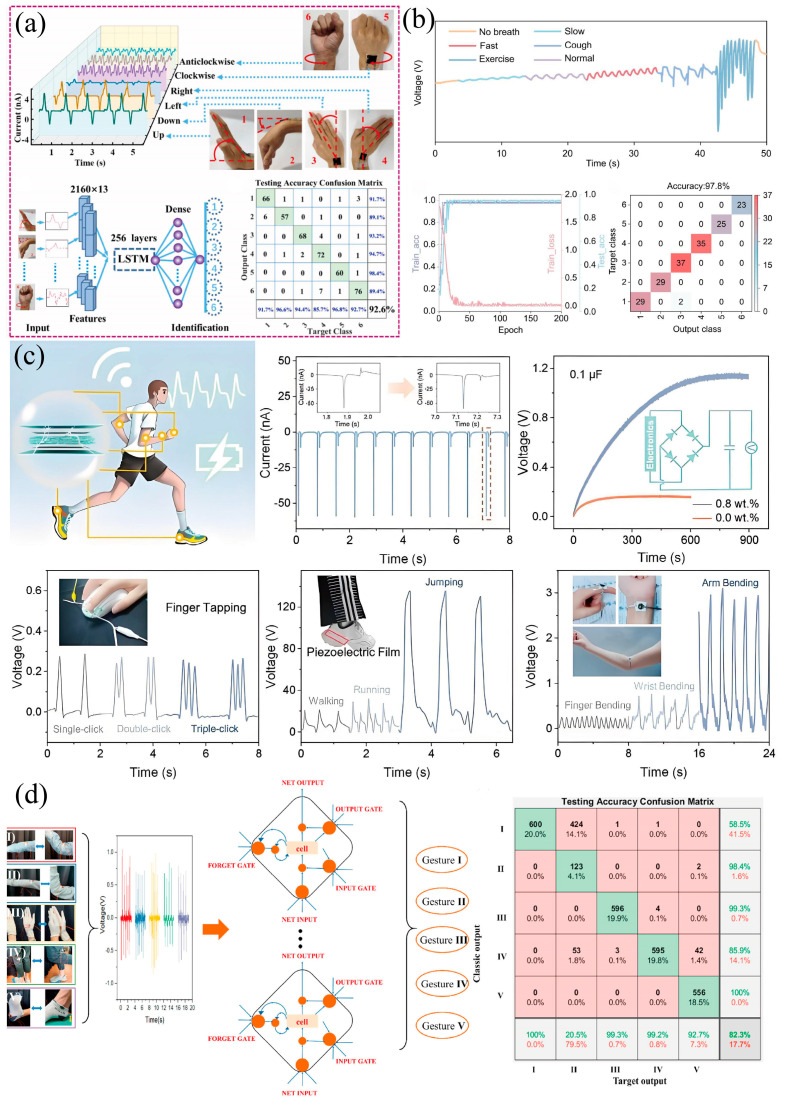
(**a**) The 3D plot of the SPP output signals and the LSTM map for six wrist motions [105]. (**b**) Breath monitoring by PSM and machine learning recognition results [104]. (**c**) Application of MXene/PVDF pressure sensor for detecting human body movements, including finger, wrist, arm, and foot sole movements [31]. (**d**) Monitoring of different hand gestures by BHSS pressure sensor, including elbow bending, clapping, leg raising, and stepping [115].

A PVDF-based flexible pressure sensor is utilized as the core of a wearable health monitoring device, which mainly collects critical physiological signals such as pulse waves, respiration, vocal cord vibration, etc. According to the collected signals, it can be used for the monitoring and prevention of human physiological diseases [116,117,118]. Guo et al. [105] proposed a self-powered piezoelectric sensor patch (SPP) combined with deep learning, with the test accuracy of SPP in wrist motion recognition reaching 92.6%, showing great application potential in sports monitoring, medical diagnosis, and rehabilitation training. As shown in Figure 9a, the SPP pressure sensor was applied to detect the signal of the wrist joint. Lan et al. [101] developed a high-sensitivity coaxial nanofiber face mask (PSM) for machine-learning-assisted breathing monitoring (Figure 9b). The mask uses the coaxial composite structure of PVDF and carbon nanotubes (CNT) to achieve a high sensitivity of 3.7 V N^−1^ and a fast response time of 20 ms, which can accurately identify multiple breathing patterns with a classification accuracy of up to 97.8%.

### 3.2. Motion Monitoring

In motion monitoring, PVDF piezoelectric sensors can sense the pressure or strain generated by human movement and convert it into electrical signals for output. These electrical signals, after subsequent processing and analysis, can provide precise information about the state of motion. Thus, PVDF-based flexible pressure sensors are also widely used in the field of sports, and the monitoring of human motion is very important to ensure the correct posture during exercise. Zhang et al. [31] successfully prepared high-performance self-powered piezoelectric sensors by spatially confined MXene/PVDF nanofibers, which significantly enhanced the monitoring resolution of human motion (Figure 9c). The PVDF-based pressure sensors are integrated with the wireless circuit system and Bluetooth module to realize the wireless monitoring and analysis of human motion [72].

### 3.3. Human–Machine Interaction (HMI)

The PVDF flexible pressure sensor also has a good application for gesture recognition. Syu et al. [114] prepared the flexible pressure sensor by the electrospinning PVDF nanofibers for multifunctional pressure sensing and human gesture recognition (Figure 9d). Through deep learning methods, especially long short-term memory networks (LSTM), the recognition of five human body gestures was realized, and the classification accuracy reached 82.3%. Similarly, Gao et al. [119] designed a tetrapod zinc oxide/PVDF (T-ZnO/PVDF) intelligent glove to realize real-time gesture recognition by finger movement.

### 3.4. Smart Home

**Figure 10:**
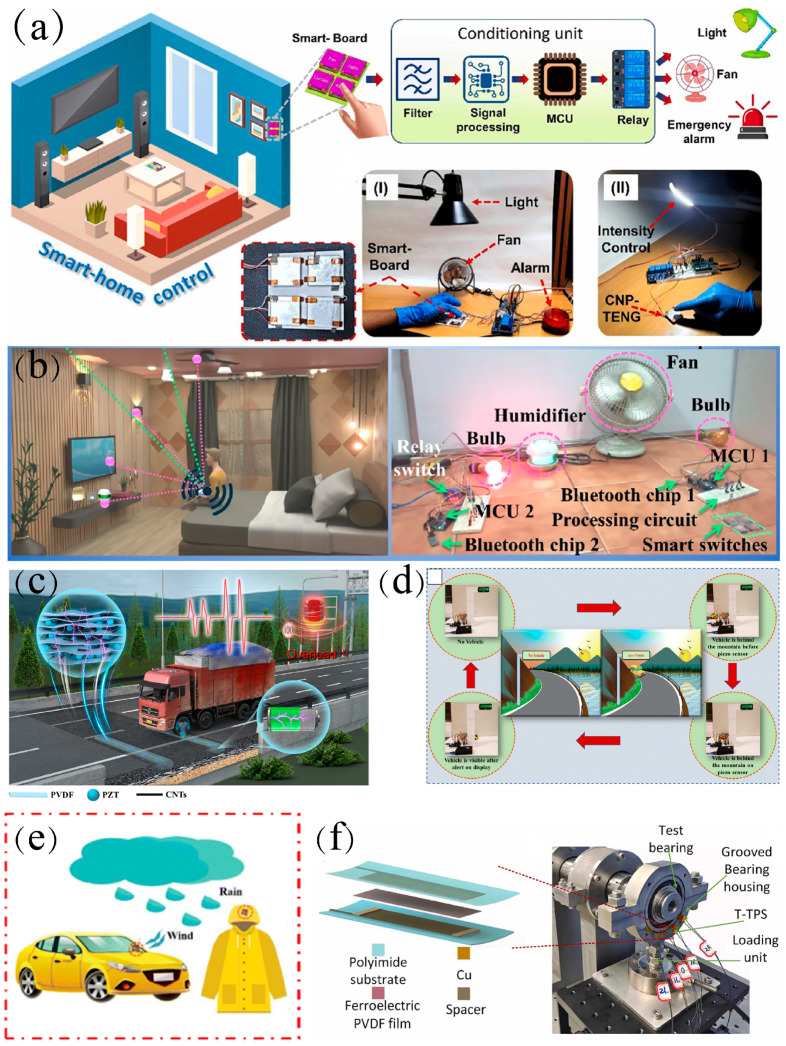
(**a**) Schematic of controlling smart home appliances using CNP-TENG-based smart board and (I) real-time smart home control system and (II) application demonstration photographs of intensity control of LED lamp using CNP-TENG [32]. (**b**) Schematic of real-time control of smart home and digital photographs of real-time wireless control of smart home appliances by the smart switch assembled by TEH10 [120]. (**c**) Schematic of PVDF/PZT/CNTs composite structure and piezosensor for road traffic information sensing, monitoring, and energy harvesting [124]. (**d**) Schematic and photograph of laboratory scale setup to illustrate the prototype piezosensor at a dangerous road turn [125]. (**e**) Schematic of ZnO@CF/PVDF composite PNG for application in outdoor clothing and vehicles during heavy wind and rainy days [126]. (**f**) Structural diagram of T-PPS integrated with bearing and schematic of installation of T-PPS [127].

Flexible sensors can act as the control center of smart homes, linking with other intelligent devices to realize intelligent family life. Rahman et al. [32] proposed a nanofiber (NFs) composited by MOF-derived cobalt-based nanoporous carbon (Co-NPC) and PVDF. Co-NPC has a high specific surface area and excellent nano-porosity, which greatly improves the electroactive β-phase formation of PVDF composite nanofibers. With an ultra-high sensitivity of 6.39 V kPa^−1^, it can be used as a self-powered pressure sensor for smart home control systems (Figure 10a).

Kar et al. [120] studied a flexible sensing device based on agriculture waste RHA/PVDF. The silica (SiO_2_)-rich RHA effectively altered the microstructure of PVDF and effectively enhanced the electroactive phase fraction. Due to its high dynamic pressure sensitivity, it can be used to assemble self-powered smart sensors for smart homes and libraries (Figure 10b). This sustainable bioorganic device with filler biodegradability, device processability, excellent biomechanical energy harvesting ability, and efficacy for IoT applications paves the way for efficient, flexible, self-powered electromechanical systems for next-generation smart artificial intelligence (AI) and Internet of Things (IoT) applications.

### 3.5. Other Applications

To achieve real applications in fields such as human health monitoring, sports fitness, and human–machine interaction, higher low-frequency stress/strain sensing capability and cyclic stability are required. In addition to the application scenarios mentioned above, PVDF pressure sensors are also used in underwater pressure [121] and flow velocity monitoring [122], electronic skin [123], road traffic information collection [124,125] (Figure 10c,d), as well as meteorological monitoring (Figure 10e) [126] and load distribution monitoring of bearings (Figure 10f) [127].

## 4. Conclusions

PVDF has attracted widespread attention due to its excellent piezoelectric properties, good flexibility, high mechanical strength, and low cost, among other factors, thus presenting broad application prospects in wearable electronics. This paper reviews the research progress of PVDF-based pressure sensors in recent years, mainly discussing the addition of fillers, structural design, and newly developed applications. By selecting appropriate fillers and rational structural design, the content of the β-phase can be increased, thereby improving the performance of the pressure sensor. The miniaturization, multifunctionality, wider detection range, and lower detection limit of PVDF-based pressure sensors remain the future development directions.

At the same time, how to integrate PVDF-based pressure sensors with current systems is another mainstream direction for development in the near future. As the digital era evolves, there are demands for PVDF-based pressure sensors to integrate with other new technologies, including virtual reality technology, big data, etc. Especially in the field of human–machine interaction, PVDF, due to its unique flexibility and excellent electromechanical conversion capabilities, can not only achieve a flexible perception of reality but also simulate virtual tactile feedback, greatly enhancing the interactivity of the virtual world. It can be predicted that, as their performance improves, PVDF pressure sensors will have a wider range of applications.

Although research on PVDF pressure sensors has made great progress, some critical issues need to be addressed to meet the application requirements:

(1) We generally attribute the intrinsic performance optimization of PVDF-based pressure sensors to the increased β-phase content, but the correlation between performance and the β-phase content needs to be further resolved.
(2) The precise monitoring of minor and low-frequency stress/strain in the human body is mandatory for wearable electronics as human motion is usually located in the low-frequency region.
(3) The rational and effective integration of PVDF pressure sensors with current systems serves as the core and prerequisite for true applications.
